# Case Report: Distinctive features of cognitive dysfunction and amelioration by antiseizure medication in neuronal intranuclear inclusion disease

**DOI:** 10.3389/fnins.2026.1734078

**Published:** 2026-02-04

**Authors:** Hiroya Ohara, Masami Yamanaka, Fumi Okada, Aiko Okazaki, Marisse Dy Dizon, Ajay Elangovan, Harysh Winster Suresh Babu, Mahalaxmi Iyer, Balachandar Vellingiri, Masako Kinoshita

**Affiliations:** 1 Department of Neurology, Minaminara General Medical Center, Yoshino, Nara, Japan; 2 Department of Clinical Laboratory, Minaminara General Medical Center, Yoshino, Nara, Japan; 3 Department of Pathology and Diagnostics, Nara Medical University School of Medicine, Kashihara, Nara, Japan; 4 Department of Dermatology, Minaminara General Medical Center, Yoshino, Nara, Japan; 5 Department of Neuroscience and Behavioral Medicine, University of Santo Tomas Hospital, Manila, Philippines; 6 Human Cytogenetics and Stem Cell Laboratory, Department of Zoology, Central University of Punjab, Bathinda, Punjab, India; 7 Department of Microbiology, Central University of Punjab, Bathinda, Punjab, India; 8 Department of Neurology, National Hospital Organization Utano National Hospital, Kyoto, Japan

**Keywords:** agraphia, antiseizure medications, cognitive dysfunction, dementia, neuronal intranuclear inclusion disease, nonconvulsive status epilepticus, skin biopsy, visuospatial execution

## Abstract

**Aim:**

Neuronal intranuclear inclusion disease (NIID) is a rare neurodegenerative disease. We investigated a role of nonconvulsive status epilepticus (NCSE) in cognitive dysfunction in pathologically confirmed NIID. We also analyzed distinctive factors to differentiate NIID from Alzheimer’s disease (AD).

**Methods:**

A 63-year-old man presented with transient consciousness disturbance (Day 1). For previous 6 years he had been suffering from similar episodes and gradually progressive cognitive decline. Clinical characteristics and response to antiseizure medications (ASM) were analyzed with a narrative literature review.

**Results:**

Neurological examination showed disorientation, memory disturbance, aphasia, agraphia, and impaired visuospatial ability. On Day 27, his MMSE scored 10. Diffusion-weighted MRI showed high intensity signal in the corticomedullary junction of the frontal lobe, which could not explain his neurological manifestations. EEG showed seizure patterns arising from the bilateral occipital areas. ASM improved MMSE score to 23. Skin biopsy confirmed his diagnosis as dementia-dominant sporadic NIID. He died on Day 77. Cognitive dysfunction in visuospatial execution, manifested by impaired pentagon drawing and agraphia of both kanji (Japanese morphograms) and kana (Japanese syllabograms), its fluctuating course, and reactivity to ASM were clear distinction from AD.

**Conclusion:**

NCSE can accelerate cognitive decline and ASM can improve cognitive function in NIID. Cognitive evaluation using pentagon drawing and handwriting of both morphograms and syllabograms can be useful to differentiate NIID from AD.

## 1 Introduction

Neuronal intranuclear inclusion disease (NIID) is a rare neurodegenerative disease characterized by widespread eosinophilic hyaline intranuclear inclusions throughout the body (Lindenberg et al., 1968; Sone et al., 2016). Patients with NIID present with pyramidal and extrapyramidal symptoms, cerebellar ataxia, dementia, and epileptic seizures (Sone et al., 2016). Since utility of skin biopsy for the antemortem diagnosis of NIID was reported (Sone et al., 2011), there are growing number of cases diagnosed as NIID. Recently, GGC repeat expansion in *NOTCH2NLC* gene was found in NIID (Sone et al., 2019). However, no effective therapy for NIID has been established yet. Several reports showed patients with NIID manifesting with new-onset epilepsy or generalized convulsion due to convulsive status epilepticus in the elderly (Toyota et al., 2015; Yamanaka et al., 2019), but characteristics and mechanism of epileptic seizures of NIID still remain unclear.

Nonconvulsive status epilepticus (NCSE) can cause cognitive decline. It has been determined that about 16% of patients aged 60 or older with confusion of unknown origin have NCSE (Cheng, 2014). Status epilepticus (SE) can be associated with later pathological processes including neuronal loss and gliosis in the hippocampus, causing chronic epilepsy and cognitive decline (Walker, 2018; Qiu et al., 2020). A previous case report described a patient with NIID who developed recurrent NCSE (Shindo et al., 2019). Thus, cognitive decline of NIID can relate to NCSE and can possibly be, at least partially, treatable.

In this study, we investigated a role of NCSE in cognitive dysfunction and effect of antiseizure medications (ASMs) in pathologically confirmed NIID. We also analyzed distinctive factors to differentiate NIID from Alzheimer’s disease (AD) using a narrative literature review.

## 2 Case description

**Figure 1:**
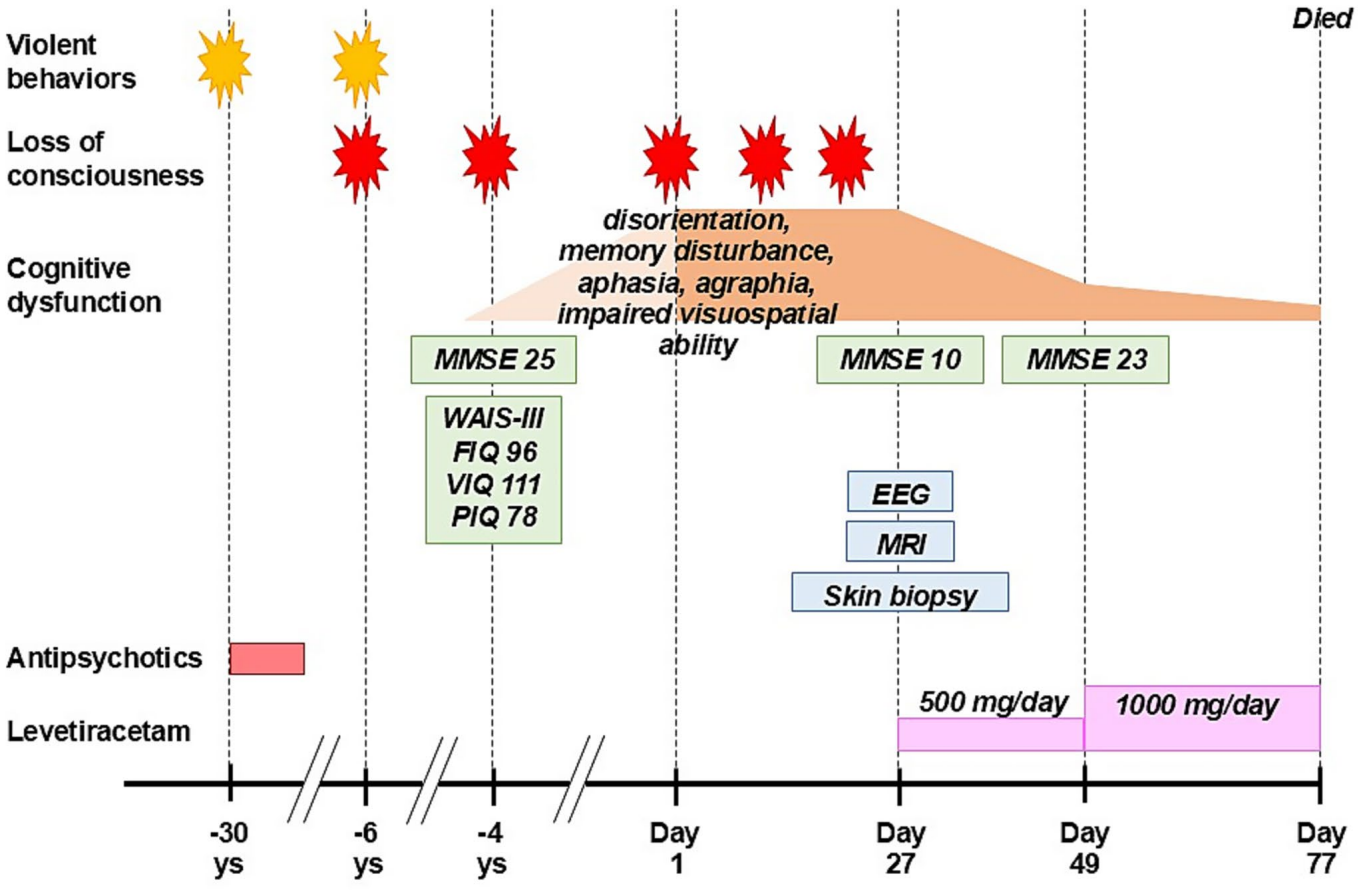
Clinical course. MMSE, The Mini-Mental State Examination; WAIS-III, The Wechsler Adult Intelligence Scale 3rd edition; FIQ, full scale intelligence quotient; VIQ, verbal intelligence quotient; PIQ, performance intelligence quotient; EEG, electroencephalography; MRI, magnetic resonance image.

A 63-year-old right-handed Japanese man, who presented with recurrent episodes of loss of consciousness with retrograde amnesia and progressive cognitive dysfunction, was investigated (Figure 1). He visited our hospital to clarify the cause of his loss of consciousness which caused his third traffic accident a day before (Day 1). He was not aware of the episode. In his 30s he was violent towards his wife and divorced 10 years later. In those times, he visited a psychiatrist and was treated with antipsychotic drugs for several years but quitted after the divorce. He changed jobs many times because of troubles with his colleagues. Six years before he had his first traffic accident, but he could not recall the whole event.

Four years before, he had femoral bone fracture due to his second traffic accident and visited our hospital. He could not recall the accident at all. In addition, he could not recognize his brother’s face for a few days. He was suspected of AD because brain magnetic resonance imaging (MRI) showed atrophy of the bilateral hippocampi (Supplementary Figure S1). Diffusion-weighted image (DWI) showed high intensity signal in the corticomedullary junction and swelling of the right temporo-occipital region, but these findings were not considered as abnormal. He was not administered any drugs and no follow-up studies were planned.

He was a social alcohol drinker and never-smoker, and had no history of hypertension, hyperlipidemia, or diabetes mellitus. No other members of his family had neurological disorders, and no consanguineous marriage was detected in his family tree.

During follow-up he showed a fluctuating course of cognitive impairment with repeated episodes of loss of consciousness. Based on the confirmation of neuronal inclusion bodies on skin biopsy from the abdominal region and findings of electroencephalogram (EEG) consistent with electrographic SE, he was diagnosed as NIID complicated with NCSE. His cognitive function improved by treatment with levetiracetam, however, he died of unknown cause on Day 77. Autopsy was not done.

In this study, we thoroughly investigated his clinical course, cognitive test results, and response to ASM, to characterize clinical presentations of NIID which could be practically utilized to differentiate from AD.

This study was carried out in accordance with the Code of Ethics of the World Medical Association (Declaration of Helsinki), and the case report was prepared in compliance with the CARE guidelines. Approval of the study design was waived by the Ethics Committee of Minaminara General Medical Center based on its classification as a retrospective analysis of anonymized clinical data. Written informed consent was obtained from the family member for the publication of the case report and any accompanying images. Part of this study was presented in abstract form at the XXVII World Congress of Neurology in 2025, held in Seoul.

## 3 Diagnostic assessment

**Figure 2:**
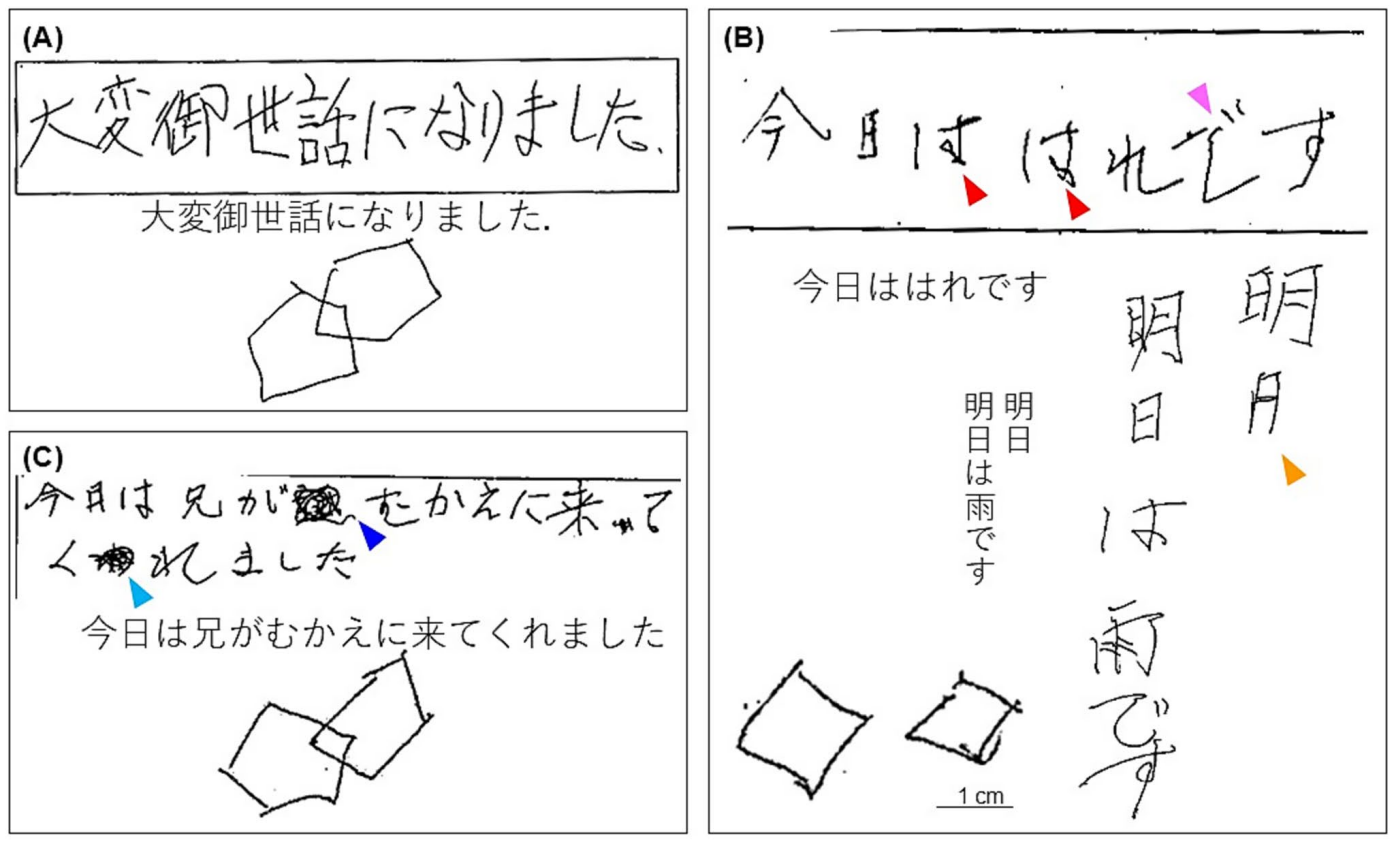
The free sentence writing test and pentagon copy test in the Mini-Mental State Examination **(A)** 4 years before, **(B)** on Day 27, and **(C)** on Day 49. **(A)** He could smoothly write both kanji (Japanese morphograms) and kana (Japanese syllabograms) and draw pentagons. **(B)** He could not write a circled part of “は” (kana) with one stroke and scribbled similar shapes (red arrowheads) nor write a bent part of “で” (kana) with one stroke and added imbalanced curve (pink arrowhead). He omitted a horizontal bottom bar of “日” (kanji) (orange arrowhead) and wrote from the beginning again. Pentagons became two separated squares. **(C)** He could write a sentence and draw pentagons, though he used “むかえ” (kana) instead of “迎え” (kanji) (the same phonemes) (blue arrowhead) and made a mistake to write “わ” (kana) which was corrected to “れ” (kana) by himself.

Four years before, the Mini-Mental State Examination (MMSE) scored 25 (orientation: −3, attention/calculation: −1, and recall: −1) (Figure 2), and the Wechsler Adult Intelligence Scale 3rd edition scored full scale intelligence quotient (IQ): 96, verbal IQ: 111, and performance IQ: 78.

On Day 1, neurological examination showed disorientation, memory disturbance, aphasia, and agraphia. Blood tests including complete blood cell count, blood chemistry, ammonia level, hemoglobin A1c, thyroid function, vitamin B1, B12 and folate, antinuclear antibody, and tumor markers were unremarkable. Serum N-terminal pro-B-type natriuretic peptide: 290.80 pg./mL (normal upper limit: 125.00 pg./mL) and soluble interleukin-2 receptor: 531 U/mL (normal range: 157–474 U/mL) were increased but no further investigation was performed. His head computed tomography was unremarkable.

**Figure 3:**
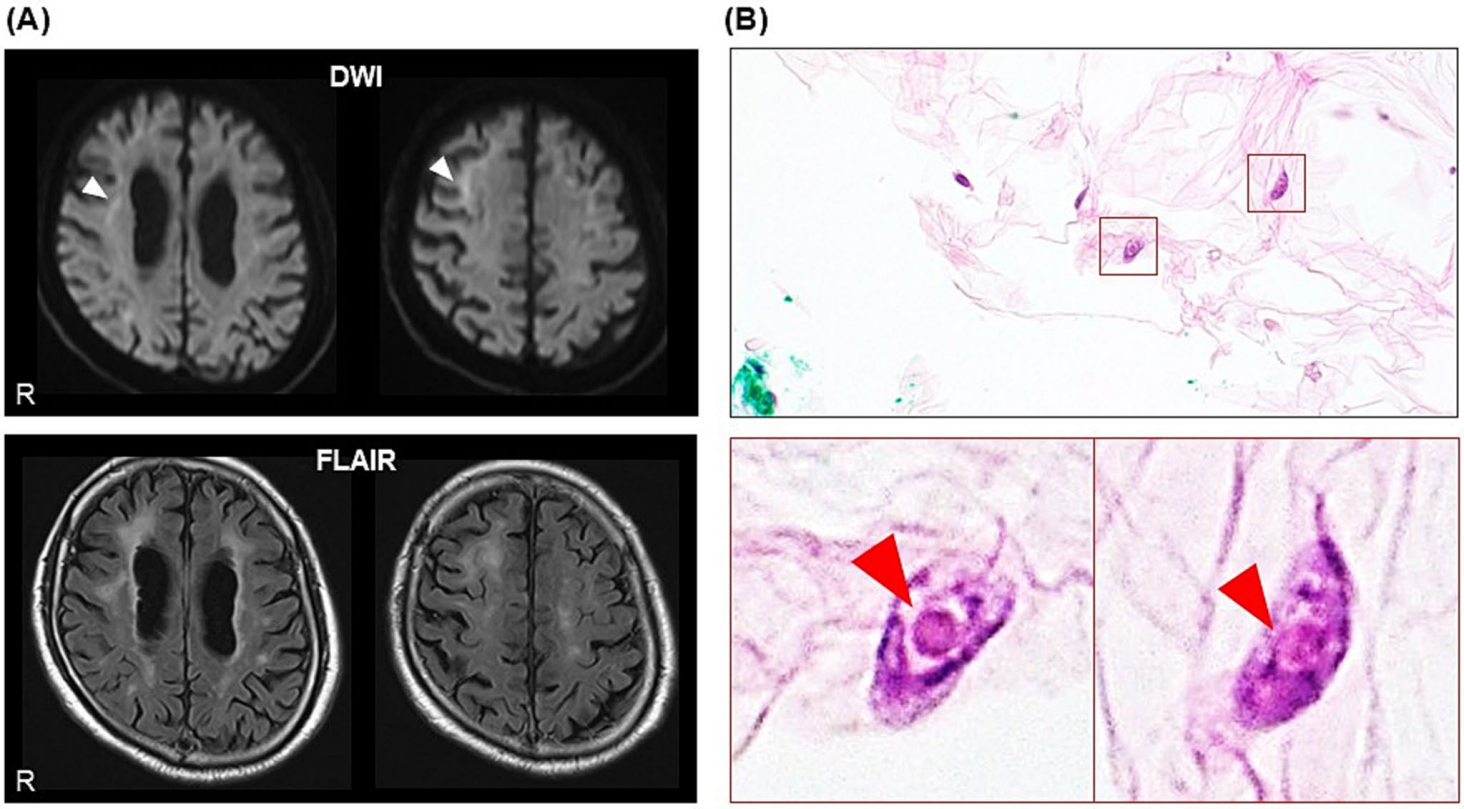
Magnetic resonance images of the brain on day 27. **(A)** Diffusion-weighted image (DWI) and fluid attenuated inversion recovery (FLAIR) image, showing high intensity signals in the corticomedullary junction, prominent in the right frontal lobe (white arrowheads). **(B)** Skin biopsy on H&E stain, showing eosinophilic intranuclear inclusion bodies in the subcutaneous adipocytes (red arrowheads).

**Figure 4:**
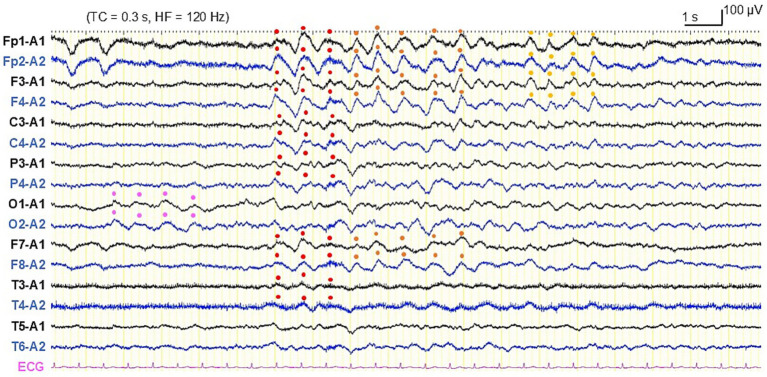
Electroencephalography on day 27. Rhythmic slow activities arising from bilateral occipital areas (pink dots), generalized (red dots), and continuing in bilateral frontotemporal areas (orange and yellow dots) with evolution in location. According to the American Clinical Neurophysiological Society’s Standardized Critical Care EEG Terminology, 2021 version, the finding is classified to electrographic seizure. With the presence of clinical manifestations for more than 10 min that were responsive to antiseizure medication, the patient was diagnosed as electroclinical status epilepticus.

On Day 27, he showed subacute progression of cognitive impairment. He had two more episodes of loss of consciousness and became unable to calculate money nor understand the notion of money. His MMSE score worsened to 10 (orientation: −9, attention/calculation: −5, recall: −3, repetition: −1, sentence: −1, and visuospatial ability: −1) (Figure 2), characterized by preservation, agraphia, and impairment of visuo-constructional ability and recent memory. He presented right-dominant postural tremor. A brain MRI showed high intensity signal in the corticomedullary junction in DWI (Figure 3A). An EEG showed almost continuous rhythmic delta activities arising from bilateral occipital areas that spread to generalized theta-delta maximum at bilateral frontal areas with evolution in frequency and distribution (Figure 4). The findings were consistent with electrographic SE according to the American Clinical Neurophysiology Society’s Standardized Critical Care EEG Terminology: 2021 version (Hirsch et al., 2021). Skin biopsy from the abdominal region, stained with H&E, confirmed neuronal inclusion bodies in the subcutaneous fat pad (Figure 3B). Other staining methods including ubiquitin and p62 and electron microscopy were not performed. According to high diagnostic significance of skin biopsy, the GGC repeat expansion was not analyzed (Sone et al., 2016; Zhao et al., 2022). AD-related biomarkers, such as Aβ, total- and phosphorylated-tau analysis in the cerebrospinal fluid or blood, amyloid PET, or FDG-PET were not conducted.

The clinical course together with imaging and biopsy findings led us to consider dementia-dominant NIID complicated with NCSE as the primary diagnosis, though the possibility of concomitant AD or other neurodegenerative disorders could not be fully excluded. He was treated with levetiracetam. On Day 49, his MMSE improved to 23 (orientation: −3, attention/concentration: −2, and recall: −2) (Figure 2). We could not evaluate a follow-up EEG before he died.

## 4 Discussion

Our sporadic case with dementia-dominant NIID showed repeated episodes of loss of consciousness and progressive decline of various cognitive functions characterized by unawareness of illness, memory disturbance, aphagia, and impairment of visuospatial ability. Interestingly, our case regained cognitive ability after starting levetiracetam. To the best of our knowledge, this is the first case with NIID who demonstrated MMSE score improvement by ASM. The current findings shed new light on role of epileptic activities in cognitive disfunction of NIID.

### 4.1 Distinctive features of cognitive dysfunction

While the patient showed cognitive dysfunction suggestive of cortical damage causing decline of MMSE score, imaging findings of his brain were discordant with the clinical manifestation because his MRI showed subcortical lesions mainly in the frontal lobe rather than cortical lesions. These features of our patient were different from common pattern of impairment in dementia-dominant NIID, which is characterized by subcortical type of executive dysfunction associated with white matter damage and more obvious decline of frontal assessment battery (FAB) scores than MMSE scores (Sone et al., 2016; Zhang et al., 2022). The distinct clinical manifestation successfully led us to diagnosis of concurrent NCSE and to administration of ASM therapy.

Cognitive impairment of our patient included visuospatial execution disturbance (Figure 2). Recent studies proposed qualitative method to assess the pentagon copy test in MMSE (the Qualitative Scoring MMSE Pentagon Tests: QSPT) for differentiating dementia with Lewy body disease (DLB) and AD, based on the rationale that visuospatial execution impairment is severer in DLB than AD and that QSPT can accurately evaluate it (Caffarra et al., 2013; Mitolo et al., 2014; Beretta et al., 2019). Caffarra et al. found significant difference of total score of QSPT between DLB and AD (mean: 6.74 vs. 9.35, *p* = 0.002) (Caffarra et al., 2013). Beretta et al. analyzed relationship between QSPT score and glucose metabolism of the brain, found different pattern of visuospatial execution disturbance between DLB and AD especially in Rotation score of QSPT, and proposed that the impairment is caused by altered visuoperceptual process in the occipital cortex of DLB whereas visuospatial process in the parietal cortex of AD (Beretta et al., 2019). As per QSPT, the pentagon copy test on Day 47 of our patient scores 6 with low Rotation score (Numbers of angles: 2, Distance/Intersection: 1, Closure/Opening: 2, Rotation: 0, Closing-in: 1), which is equivalent to DLB pattern. These findings strongly suggest that our patient had more prominent damage of the occipital cortex than the parietal cortex at the time of examination. The type of impairment cannot be recognizable by FAB.

Our patient also showed agraphia with abnormal grapheme formation and character imagery deficit for both kanji (Japanese morphograms) and kana (Japanese syllabograms) (Figure 2). He wrote sentences by trial and error. No phonological error was observed. It is known that a lesion in the superior parietal lobule is responsible to apraxic agraphia exhibiting impairment orthographic production (Alexander et al., 1992). Since the Japanese language is comprised of two writing systems of kanji and kana, precise evaluation of agraphia can be utilized to determine site of brain lesion (Iwata, 1984). More recent studies in Japanese patients with a lesion involving the superior parietal lobule reported that features of agraphia are variable according to extension of lesions (Dejerine, 1891; Kawamura, 1990; Crary and Heilman, 1988; Sakurai et al., 2000, 2007, 2008, 2010; Sakurai, 2011). With involvement of the posterior area of the superior parietal lobule alone or around the intraparietal sulcus, agraphia is significantly severer in kanji than kana. Additional damage to the angular gyrus also yields agraphia severer in kanji than kana but phonological errors (literal paragraphia) are observed, and further extension to the middle occipital gyrus causes concurrent alexia. When the posterior-medial portion of the superior parietal lobule (the precuneus) is severely affected and the superior occipital gyrus is involved as well, both kanji and kana orthography are impaired. Involvement of the supramarginal gyrus can cause agraphia including literal paragraphia for kana severer than kanji. Thus, agraphia of our patient strongly suggests the association with the superior parietal lobule, especially the precuneus, and the superior occipital gyrus.

### 4.2 Epileptic seizures and status epilepticus in NIID

Our case manifested with recurrent episodes of loss of consciousness and evaluation of possible epileptic seizures enabled us to detect NCSE. However, diagnostic significance of concurrent epileptic seizures in management of NIID has not been established yet. Shindo et al. reported a case of pathologically confirmed NIID who developed NCSE after recurrent symptoms of paroxysmal nausea and slowly progressive cognitive decline (Shindo et al., 2019). EEG of this case showed generalized periodic discharges. Effect of ASMs was not described.

Sone et al. analyzed clinicopathological features of Japanese NIID and reported that, in sporadic dementia-dominant NIID cases, 13.2% showed generalized convulsions and 39.5% showed episodes of consciousness disturbance lasting for few hours to several days (Sone et al., 2016). Moreover, 21% of the patients showed subacute encephalitic episodes exhibiting fever, headache, vomiting, and disturbance of consciousness. In Japan and Taiwan, GGC repeat expansion of *NOTCH2NLC* is revealed to be one of the major causes for adult-onset leukoencephalopathy (Okubo et al., 2019; Liao et al., 2023; Liu et al., 2022). A study in Taiwan which diagnosed 34 NIID patients reported that 19 (55.9%) patients showed acute encephalitis-like episodes (Liu et al., 2022). Of the 10 patients whose MRI was obtained, half showed restricted diffusion mainly in the posterior cerebral cortex, and focal edema of the brain was detected in 2 patients who manifested with recurrent generalized seizures. Another case report describes a middle-aged woman who presented with severe headache and agitative psychiatric symptoms. Her MRI showed swelling in the temporal and occipital lobes with contrast enhancement but no typical hyperintensities were detected on DWI (Shen et al., 2024). There is no information on EEG. These stereotyped episodes resemble to the symptoms of our patient and the previous case (Shindo et al., 2019). Precise EEG evaluation and ASM trial will reveal the role of epileptic seizures and SE, both convulsive and nonconvulsive ones, in episodic symptoms as well as in gradual cognitive decline in NIID.

It is of note that NCSE can exhibit disturbance of higher cortical function including visuospatial dysfunction as manifestation of epileptic seizure itself. A previous case with SE showed symptoms of transient diffuse right hemisphere dysfunction including hemi-spatial neglect, dyscalculia, disturbance of spatial construction, and disturbance of visuospatial perception (Vanhatalo et al., 2003). Ikeda et al. reported patients with various ictal inhibitory motor manifestations and postulated their association with apraxia (Ikeda et al., 2009). In addition, SE including NCSE results in obvious neuronal death in the hippocampus especially in the CA3 and CA1 regions, and pathological processes and behavioral change occur over several months (Walker, 2018; Qiu et al., 2020). A rodent model study showed that neuronal loss, continuous immune reaction, and alterations in excitatory and inhibitory synaptic proteins develop within 1 month following NCSE (Avdic et al., 2018).

Since we could confirm striking effect of ASM in our patient, no further intervention was performed. However, especially when seizures are refractory, etiologies should be elucidated along with full exclusion of possible comorbidities. Among patients with NIID who showed encephalitic symptoms, focal brain edema with gadolinium enhancement was found, and high dose intravenous steroid therapy was effective to them though transiently (Sone et al., 2016). Recently, new onset refractory status epilepticus was proposed as a clinical presentation to determine consensus for management (Hirsch et al., 2018). After excluding infectious etiology, first-line and second-line immunotherapy is recommended in addition to seizure suppression using ASM and anesthesia (Sheikh and Hirsch, 2023). Besides, systematic autoimmune diseases and autoimmune encephalitis should be evaluated (Graus et al., 2016). Paraneoplastic neuronal syndrome can manifest with encephalitis and cognitive decline, which require whole body evaluation for malignant tumors (Graus et al., 2016, 2021).

### 4.3 Diagnostic value of EEG for SE and NCSE

Diagnostic significance of epileptiform discharges, namely spikes and sharp waves, is still limited, mainly because of extremely low sensitivity. In adults who developed first unprovoked seizures, interictal epileptiform discharges can predict seizure recurrence with only 17.3% sensitivity whereas 94.7% specificity (Bouma et al., 2016). An epileptiform discharge represents not only excitatory but inhibitory neuronal mechanisms, thus can be invisible if these systems are severely disrupted (de Curtis and Avanzini, 2001). Ictal EEG can start with low voltage fast activities with spaciotemporal evolution without overt epileptiform discharges (de Curtis and Gnatkovsky, 2009). EEG in SE and NCSE is more complicated because, in addition to seizure activities, it also reflects brain abnormalities caused by prolonged seizure and underlying diseases which is provoking seizures.

An importance of concurrent rhythmic fast activities was described as periodic lateralized epileptiform discharges plus (Reiher et al., 1991). Thereafter, as data on critical care EEG are accumulated, diagnostic significance of rhythmic and periodic discharges in SE and NCSE is updated. The American Clinical Neurophysiology Society proposed a series of terminology to describe EEG findings in neurocritical care based on the Salzburg EEG criteria and defined electrographic and electroclinical criteria for epileptic seizures and SE (Hirsch et al., 2021). EEG of our patient during cognitive deterioration showed conventional seizure pattern with evolution exceeding 10 s almost continuously for more than 10 min, which fulfilled with the criteria for electrographic SE. Moreover, his clinical symptom dramatically improved by ASM, that fulfilled with the criteria for electroclinical SE. Although follow-up EEG could not be recorded during his lifetime, generally it is recommended to confirm improvement in EEG findings after treatment.

### 4.4 Role of epileptic seizures and ASM in management of dementia

Close relationship between epilepsy and dementia has already established (Gowers, 1885; Noebels, 2011; Sen et al., 2018). AD has an extremely high comorbidity with epilepsy because neuronal hyperexcitability induces A*β*, and both clinical and subclinical seizures progress cell death and cognitive deterioration in a vicious circle. However, focal seizures of AD are still underdiagnosed and underestimated (Mendez and Lim, 2003). Both epileptic seizures and subclinical epileptiform discharges associate with the decline of cognitive function in patients with AD (Volicer et al., 1995; Vossel et al., 2016). AD patients with seizures begin to show a decline in cognitive function 3.6 years earlier than those without seizures (DiFrancesco et al., 2017). Hippocampal interictal or subclinical epileptiform discharges can impair the maintenance and retrieval of memory (Lam et al., 2017).

There is controversy about effect of ASM on cognitive dysfunction in AD patients with epilepsy or subclinical epileptic activities. Clinical and basic studies have indicated that proper ASM potentially improve the prognosis of cognitive performance, specifically oral fluency items and attention, in AD patients with epilepsy (Volicer et al., 1995; DiFrancesco et al., 2017; Liu et al., 2018). A study in AD model mouse showed that ASM can reduce Aβ-protein levels by suppressing β-cleavage of amyloid precursor proteins (Ueda et al., 2023).

### 4.5 Limitations

This study has four main limitations. First, this is a single case study of dementia-dominant NIID, which cannot provide scientific generalization. Second, the *NOTCH2NLC* gene testing was not performed and we could not obtain GGC repeat number to analyze genotype–phenotype association. Third, we could not confirm improvement on EEG after ASM treatment. Fourth, AD-related biomarkers were not examined, and possible association with AD or other neurodegenerative disorders could not be completely ruled out. Further studies involving more cases are warranted to address these concerns.

## 5 Patient perspective

NCSE can accelerate cognitive decline, and antiepileptic drugs can ameliorate cognitive function, in pathologically confirmed NIID. In patients with atypical and fluctuating course of dementia, especially those with recurrent episodes of consciousness disturbance and encephalitic symptoms, diagnostic evaluation for NIID and SE and NCSE and ASM therapy are strongly recommended.

## Data Availability

The original contributions presented in the study are included in the article/Supplementary material, further inquiries can be directed to the corresponding author.

## Supplementary material

SUPPLEMENTARY FIGURE S1 Magnetic resonance imaging of the brain 4 years before. Diffusion-weighted image showed atrophy of the bilateral hippocampi, high intensity signals in the corticomedullary junction mainly in the bilateral frontal lobes and swelling of the right temporo-occipital region (white arrowhead).
