# Supplementary figures and images for: Case Report: Distinctive features of cognitive dysfunction and amelioration by antiseizure medication in neuronal intranuclear inclusion disease
Source: Front Neurosci. 2026 Feb 4;20:1734078. doi: 10.3389/fnins.2026.1734078 (PMC12913443; doi:10.3389/fnins.2026.1734078)

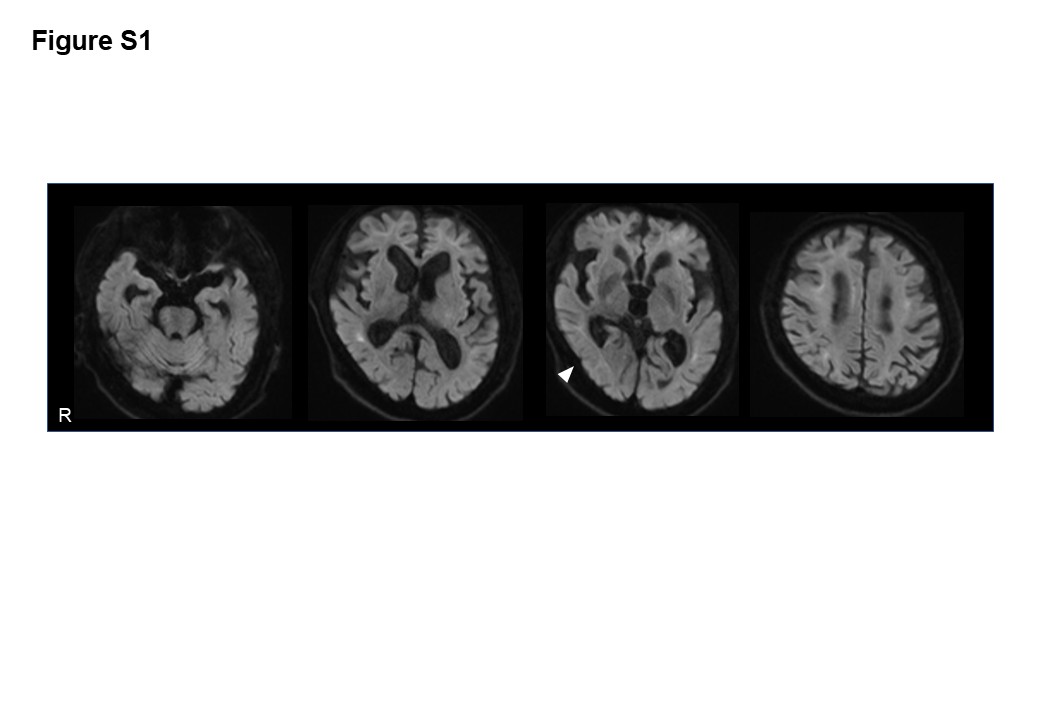

Supplement: SUPPLEMENTARY FIGURE S1 — Magnetic resonance imaging of the brain 4 years before. Diffusion-weighted image showed atrophy of the bilateral hippocampi, high intensity signals in the corticomedullary junction mainly in the bilateral frontal lobes and swelling of the right temporo-occipital region (white arrowhead). [file Image_1.JPEG]
